# The effects of technological and traditional feedback on back squat performance in untrained women

**DOI:** 10.1186/s13102-022-00556-5

**Published:** 2022-09-02

**Authors:** N. Stien, V. Andersen, G. H. Engelsrud, T. E. J. Solstad, A. H. Saeterbakken

**Affiliations:** 1 Faculty of Education, Arts, and Sports, Department of Sport, Food, and Natural Sciences, Western Norway University of Applied Sciences, Bergen, Norway; 2 Faculty of Education, Arts, and Sports, Department of Sport, Food, and Natural Sciences, Western Norway University of Applied Sciences, Sogndal, Norway

**Keywords:** Strength, Movement quality, Technique, Training

## Abstract

**Background:**

Recently, a novel method for improving movement quality called open-ended augmented feedback has been introduced. However, the effects of using such feedback in a training intervention have not yet been examined. The aim of this study was to assess the changes in performance and movement quality following a five-week resistance-training program with either (1) technological feedback or (2) traditional, verbal feedback from an experienced trainer.

**Methods:**

Nineteen untrained females (age: 21.84 ± 2.24 years, height: 169.95 ± 5.92 cm, body mass: 65.05 ± 7.93 kg) randomly allocated to one of the two conditions completed five weeks of training with two weekly sessions. Pre- and post-intervention, participants were tested for physical performance (i.e., back squat and isometric mid-thigh pull strength) and movement quality parameters (weight distribution, center of gravity variation, and subjective rating of the back squat technique).

**Results:**

Both groups similarly increased the training resistance throughout the intervention (*p* < 0.01), as well as strength in the back squat (technological feedback group: effect size (ES) = 1.31, *p* = 0.002; traditional feedback group: ES = 1.48, *p* = 0.002). Only the traditional feedback group increased isometric mid-thigh pull strength (ES = 1.11, *p* = 0.008) and subjectively rated lifting technique at the same load (*p* = 0.046). No changes in force distribution (*p* = 0.062–0.993) or center of gravity variation (*p* = 0.160–0.969) occurred in either group when lifting the same absolute loads at post-test. However, both groups displayed a greater variation in center of gravity when lifting the same relative load at post-test (technological feedback group: *p* < 0.001; traditional feedback group: *p* = 0.006). No differences were found between the groups for any of the observed changes (*p* = 0.205–0.401).

**Conclusions:**

Five weeks of back-squat training with verbal feedback increased isometric mid-thigh pull strength and subjectively rated lifting technique from pre- to post-test, whereas technological feedback did not. Both methods improved back squat strength and training resistance. For resistance-training beginners, the choice between feedback methods should be based on the desired outcomes and the availability of expertise and equipment.

## Background

Performance in resistance-training (RT) is usually quantified using the maximal external load that can be lifted for a given number of repetitions. For RT to be an effective tool for improving strength, high training quality in the form of power output and technical execution is necessary [1–3]. Verbal feedback from a trainer is a common method for optimizing and improving RT quality, and has demonstrated improved motivation, competitiveness, self-selected training load, and performance in RT [4–7].

Since the human eye may be a limited tool for detecting minor errors in the movement pattern, novel technological methods (e.g., real-time feedback on movement velocity, balance, body positioning, or force distribution) for assessing and improving the technical execution of bodily movements have been investigated in recent years [8–12]. For example, real-time feedback on movement velocity in each repetition, in the absence of a trainer, has been shown to improve back-squat performance [11]. Conversely, others have concluded that automated, technological feedback may be less effective compared to verbal feedback or a combination of the two [12]. Automated feedback may be limited by (1) the fact that correct execution must be pre-defined, (2) only being able to assess single parameters, and (3) lacking the social and communicative skills that an experienced trainer can display. Moreover, the relationship between the experience level of the trainee and the complexity of the task likely influence the usefulness of automated feedback mechanisms [9]. Hence, the human component is likely still a vital component of the feedback process.

Recently, a novel method for improving movement quality has been introduced: open-ended augmented feedback [10]. The concept is grounded in embodied cognition theory which states that learning manifests in a circular interaction between the central nervous system and bodily sensations, emotions, and perceived affordances in the environment [13, 14]. In order to emphasize this type of learning, open-ended augmented feedback in the form of, for example, lights [10] or sounds [8] is used to illustrate the outcome of the performance or execution for the trainee. The interactions between expectations, outcome, and feedback can then be used by the trainee to generate a relationship between the feeling of a movement and the respective outcome [15]. Over time, trainees can discover the most desirable movement pattern and intrinsically gravitate toward this execution. However, the longitudinal effects of open-ended augmented feedback in RT have not yet been examined.

Based on the current gaps in the knowledge about the effects of technological feedback in RT, this study aimed to address the potential changes in strength and movement quality of the back squat following RT with either (1) open-ended technological feedback using the body-lights approach introduced by Vidal and colleagues [10] and (2) traditional verbal feedback. Based on the existing knowledge, it was hypothesized that technological feedback would induce similar improvements in strength and movement quality when tested without feedback after the intervention.

## Method

A randomized parallel trial with within- and between-groups comparisons was designed to address the research question. The participants were randomized to five weeks of back-squat training with one of the following conditions: (1) open-ended technological feedback (TECH) using body-lights [10] or (2) traditional, verbal feedback from an experienced instructor (TRAD). Participants were tested for back squat and isometric mid-thigh pull strength and technical execution (i.e., balance, force distribution, and expert rating) of the back squat before and after five weeks of training with one of the following conditions.

### Participants

Twenty-two healthy, untrained females without systematic RT experience in the last eighteen months were recruited for this study. Three participants withdrew from the study due to personal reasons, while the remaining nineteen participants completed the training and testing (Table 1). The participants were informed verbally and in writing about the potential risks and benefits of participation and signed an informed consent form before data collection began. The research procedures conformed to the ethical guidelines of the university and to the standards of treatment of human participants in research, as outlined in the latest revision of the Helsinki declaration. Furthermore, the procedures were processed by the Regional Committees for Medical and Health Research Ethics (Reference 323,304) and by the Norwegian Centre for Research Data (Reference 501,802).

**Table 1:** Characteristics of the participants at baseline (mean ± SD)

	TECH (*n* = 10)	TRAD (*n* = 9)	Total (*n* = 19)
Age (years)	21.60 ± 1.96	22.11 ± 2.62	21.84 ± 2.24
Height (cm)	169.20 ± 4.47	170.78 ± 7.41	169.95 ± 5.92
Body mass (kg)	66.60 ± 8.28	63.33 ± 7.62	65.05 ± 7.93

### Testing procedures

When first arriving at the laboratory, the participants were tested for anthropometric parameters. The BM was measured using a bioelectric impedance scale (Tanita MC 780MA S, Tokyo, Japan), and height was measured using a wall-mounted measuring tape.

#### Warm-up

Before the testing commenced, a ten-minute warm-up consisting of 15, 10, and 6 repetitions of back squat using 8 kg, 20 kg, and approximately 50% of self-reported estimation of 10 repetitions maximum, respectively. If a participant was unable to estimate her 10 repetitions maximum due to little or no experience with the back-squat, the researchers suggested a load based on visual inspection of the performance in the 8 kg and 20 kg conditions, which had to be agreed upon by the participant. An identical warm-up procedure was used for all training sessions. The bottom position was defined as a 90° knee angle (measured with a goniometer during the two lightest warm-up loads). The participants were instructed to try to reach approximately this depth in all repetitions and use a self-selected, but controlled tempo throughout the set. If necessary, verbal instructions to adjust the depth were given and new goniometer measurements taken. These instructions were identical for the experimental tests.

#### Back-squat testing

Five minutes after completing the warm-up, the participants performed three sets with ten repetitions of back squat. The sets were performed using (1) only the bar (20 kg), (2) 50% of BM, and (3) a load that allowed ten repetitions to be completed with approximately three repetitions in reserve (RIR). The submaximal loads were chosen due to the low training experience of the participants. A 0–10 rating of perceived exertion (RPE) scale was used to estimate the RIR based on the methods applied by Zourdos and colleagues [16], suggesting that an RPE of seven would correspond to three RIR. Unbeknownst to the participants, the testing was terminated when they reported an RPE of seven or higher (reached within two attempts for all participants). If an RPE of six or less was reported, the load was increased by 2.5–5 kg for the next set. The same three absolute loads were lifted at post-test (20 kg, 50% BM, and the RPE ≥ 7 load). Importantly, the increased strength and familiarization with the exercise reduced the RPE at any given absolute load. Indeed, both groups reported lower RPEs after lifting the same absolute RPE ≥ 7 load at post-test compared to pre-test (*p* = 0.004 and *p* = 0.009 for the TECH and TRAD groups, respectively). Therefore, a fourth condition (RPE_post_) was included at post-test using a similar level of effort (i.e., RPE ≥ 7) as the third set at pre-test. In correspondence with the pre-testing procedures, the loads were gradually increased until terminating the set when an RPE ≥ 7 was reported. The highest load lifted for ten repetitions with approximately three RIR at pre- and post-test was used as a measure of maximal strength (3_RIR_). The reported RPEs for the tech group were 7.6 ± 0.7 at pre-test and 7.6 ± 0.5 at post-test (*p* = 1.000), whereas the TRAD group reported RPEs of 8.1 ± 0.9 and 8.4 ± 0.9 (*p* = 0.830), respectively. All testing was conducted without any form of feedback or verbal encouragement.

During the experimental sets, weight distribution between the legs was assessed using two independent force plates [3] with a resolution of 200 Hz (Ergotest Innovation A/S, Porsgrunn, Norway) that were connected to a computer with the commercial software MuscleLab V10.4 (Ergotest Innovation A/S, Porsgrunn, Norway). The force plates were placed eight centimeters apart for all participants, but the positioning of the feet (angle and distance between feet) was registered at pre-test and the identical position was used a post-test. The force distribution between the legs was collected from the average of the first and second repetitions and the ninth and tenth repetitions. The force distribution was calculated as (((dominant leg force–non-dominant leg force)/total force)*100) [3]. The “dominant” leg was defined as the leg that produced the highest force output during the back-squat execution and this did not change from pre- to post-test for any of the participants.

The two force plates were placed on top of a third, larger force plate that registered the center of gravity variation in the X- and Y-axes. Unfortunately, when collecting data from several force plates simultaneously, the software was unable to provide the distance travelled by the center of gravity. Instead, the coefficient of variation (CV; (mean placement/standard deviation)*100) was calculated and used as a measure of the variation during first and last two repetitions.

Further, the execution of the ten repetitions was recorded simultaneously from two angles (anterior and lateral) using two Logitech web-cameras (C920 PRO, Lausanne, Switzerland) connected to a computer with the free commercial software Kinovea (version 0.9.4). The recordings were reviewed by three RT experts with Ph.D. and master’s degrees in sports science and several years of instructing and teaching experience. The experts were blinded to which testing session (pre or post) the recordings were collected and of group allocation. In addition, the participants’ faces were blurred. The experts evaluated the technical execution of the lifts and provided their scoring on a three-point scale ranging from 0 (poor/incorrect) to 2 (good/correct) which has been shown reliable for assessing lower-limb exercises [17]. Two weeks after reviewing the recordings, the expert panel reviewed the videos again in a randomized order to assess the reliability and agreement of the ratings.

#### Mid-thigh pull test

**Fig. 1:**
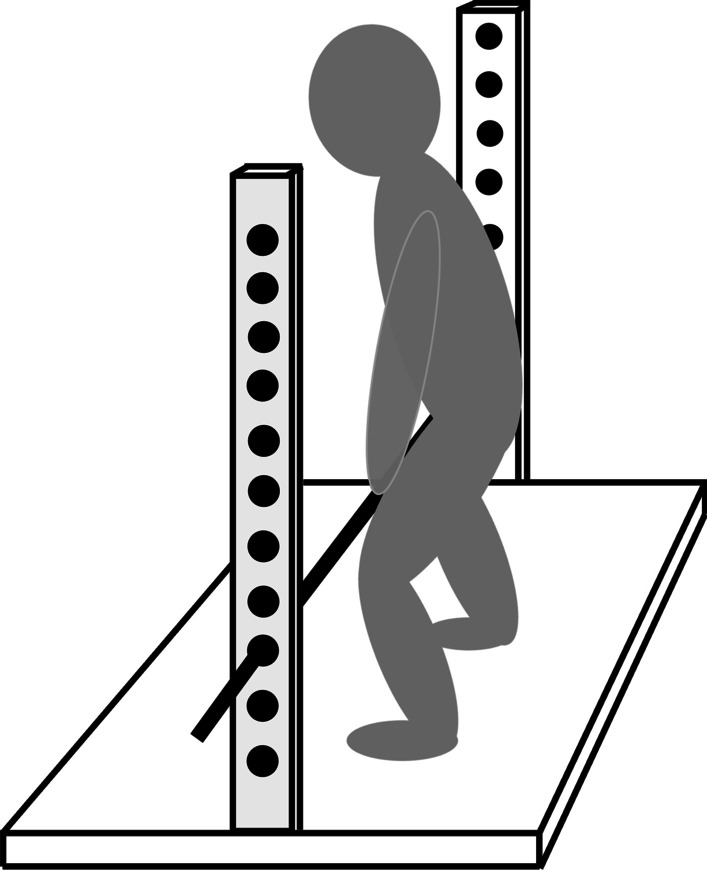
Schematic illustration of the mid-thigh pull test. Author’s own work

Finally, maximal isometric strength was assessed in the mid-thigh pull exercise using a custom-built apparatus (Fig. 1). The participants stood on a force plate with a 200 Hz resolution (Ergotest Innovation A/S, Porsgrunn, Norway) holding onto a fixed bar. The body positioning was adjusted for individual height by adjusting the height of the bar (5 cm intervals). The knee and hip angles were approximately 125° and 145°, respectively, based on previous recommendations for individuals without substantial weightlifting experience [18, 19]. The joint angle measurements were taken with the participants applying a minor force to the bar. The stance (foot width and angle) was self-selected, but registered and identical in both testing sessions. Previous recommendations for postural instructions were applied [19]. The participants were instructed to gradually increase the applied force for one-to-two seconds before exerting maximal effort for five seconds. Three acceptable trials had to be conducted with less than 10% difference in maximal average force output (F_max_; average across the three seconds with the highest force output). If a > 10% difference was detected, an additional trial was conducted. The CVs for the three trials were 2.09% and 2.99% on pre- and post-test, respectively. The F_max_ of the best attempt was registered and used in the analyses.

### Training

The participants were prescribed ten supervised training sessions over the course of five weeks (two weekly sessions), including three sets of ten repetitions. At least 80% attendance to the training was required to be included in the analyses and an average attendance of 96.3% was reached (range: 80–100%). No participants were excluded from the analyses for not fulfilling this requirement.

Each session was supervised by the same instructor and lasted around 20 min, including the warm-up. To maintain a standardized and ecologically valid training condition, a set of ques to be used during the training was developed in cooperation with seven professional personal trainers (Table 2). The ques for the TRAD group provided basic back-squat instructions. Two main ques were provided to the TECH group, as well as informing the participants about what the information from the laser pointers meant and how they could influence the movement of the laser. This information was provided in weeks one and two, whereas they were able to interpret the laser pointers independently with minimal use of instructions for the remaining duration of the training.

**Table 2:** Instructional ques used by the instructor in the training

TRAD	TECH
Try to push equally hard with both feet	Keep the dots horizontally aligned
Strive to press using the whole foot	Try to keep the dots within the vertical lines
Remember to engage the core muscles	
Maintain a slight outward knee rotation	

**Fig. 2:**
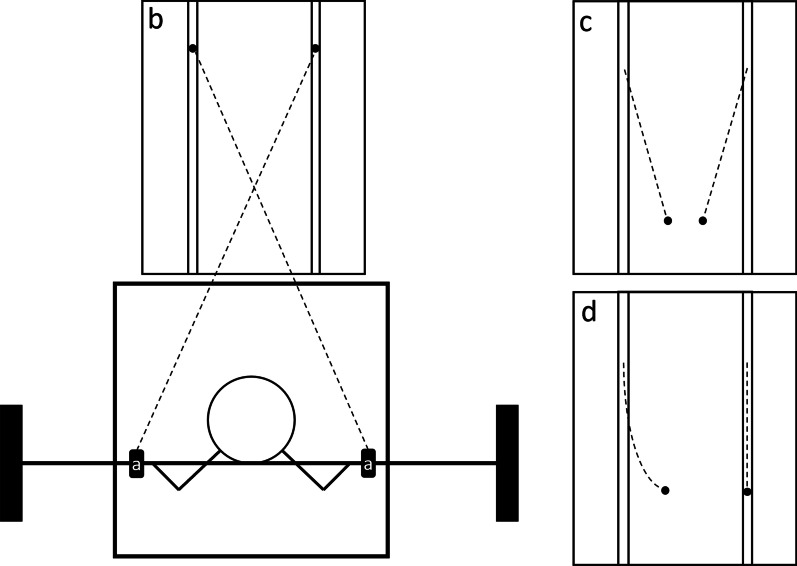
Schematic illustration of the set-up for the feedback during training for the TECH group showing **a** the attachment points of the laser pointers, **b** the whiteboard with fixed reference points, and examples of the lights’ movement when **c** tilting forward and **d** putting more pressure on one foot compared to the other

For the TECH group, two laser pointers were attached 46 cm from the center of the barbell (Fig. 2a). These were pointed slightly inwards to cross (i.e., the right pointer produced the left dot) and project to a reflective whiteboard placed 150 cm in front of the barbell with predefined reference points (Fig. 2b). In the starting position, the dots were 15 cm apart. If the participants tilted forward during the lift, the dots would move closer (Fig. 2c). Moreover, changes in weight distribution were reflected in a deviation from the linear movement trajectory by one of the dots. For example, extra pressure on the right foot and a subsequent slight rotation of the torso would cause the left dot to move medially while the right dot maintained a vertical line (Fig. 2d).

The training load during the intervention was self-selected. The participants were encouraged to increase the load throughout the intervention, but to prioritize selecting a load that they could confidently lift ten times with proper technique and approximately two RIR. Autoregulation using RIR has previously increased effort and improvement magnitude in back squat strength compared to using fixed loading [20]. Furthermore, training to failure could be problematic for a population without prior RT experience, especially in complex exercises (e.g., back squat) where poor technique may increase risk of injury [21].

### Statistical analyses

All analyses were performed using the commercial statistical software SPSS (IBM Corp. Released 2020. IBM SPSS Statistics for Windows, Version 27.0. Armonk, NY: IBM Corp). Data was assessed for normality using a Shapiro–Wilk test and none of the anthropometric or performance parameters (data collected during the back squat and isometric mid-thigh pull) displayed a deviation from a normal distribution (*p* = 0.089–0.864). Differences between the groups at pre- and post-test were assessed using analyses of covariance (ANCOVA) with the pre-test results as the covariate. Paired-samples t-tests were used to address the within-groups changes. The RPE and expert scoring were not normally distributed (*p* < 0.025 and *p* < 0.001, respectively) and were analyzed using a Wilcoxon signed rank test for the within-groups comparisons and a Mann–Whitney U test for the between-groups differences. Spearman’s rho was used to measure the intra-rater reliability, whereas the inter-rater reliability was assessed with the intraclass correlation of the absolute agreement. A Spearman’s rho of < 0.40, 0.41–0.60, 0.61–0.80, or 0.81–1.0 was interpreted as weak, moderate, strong, and very strong, respectively [22]. Intra-class correlations of 0.21–0.40, 0.41–0.60, 0.61–0.80, and 0.81–0.99 were interpreted as fair, moderate, substantial, and near perfect agreement [23]. Statistical significance was accepted at *p* < 0.05. The results are presented as means with standard deviations and Hedges’ *g* effect sizes (ES) for the changes. The ES were calculated as the mean difference divided by the pooled and weighted standard deviations. ES of < 0.2, 0.2–0.5, 0.5–0.8, and > 0.8 were interpreted as trivial, small, medium, and large, respectively [24].

## Results

### Training resistance

**Fig. 3:**
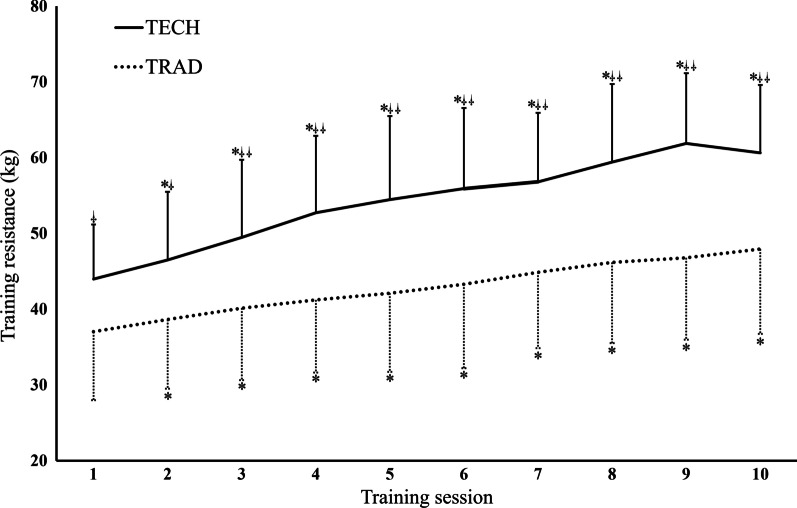
The mean training resistance in each session throughout the intervention. * = Higher training resistance than the first session (*p* < 0.01).  = Higher training resistance than the TRAD group (*p* < 0.01).  = Higher training resistance than the TRAD group (*p* < 0.001)

The average training resistance was higher for the TECH group compared to the TRAD groups in all training sessions (*p* < 0.01). All subsequent training sessions included a higher training resistance than the first session for both groups (*p* < 0.01). See Fig. 3 for an overview of the training loads used throughout the intervention for the two groups.

### Expert scoring

The correlation between the two rounds of expert ratings were significant (*p* = 0.016), but weakly correlated (r = 0.189). Regarding the inter-rater reliability displayed significant and substantial agreement across the three experts (Intra-class correlation = 0.650, *p* < 0.001).

The average expert scoring at pre-test (TECH = 1.70 ± 0.48; TRAD = 1.33 ± 0.71) was not different between the groups (*p* = 0.315). When lifting the same absolute load at post-test, the TRAD group (score = 1.89 ± 0.33; ES = 0.98, *p* = 0.046) received a higher score than at pre-test, whereas the TECH group did not (score = 1.70 ± 0.48; ES = 0.00, *p* = 1.000). The TRAD group also achieved a higher score when lifting the same relative load (same RPE) at post-test (score = 1.89 ± 0.33; ES = 0.98, *p* = 0.046), whereas the TECH group did not (score = 1.60 ± 0.52; ES = 0.20, *p* = 0.317). The changes from pre- to post-test was not different between the groups (*p* = 0.133) and the groups were not different at post-test (*p* = 0.497).

### Strength

**Fig. 4:**
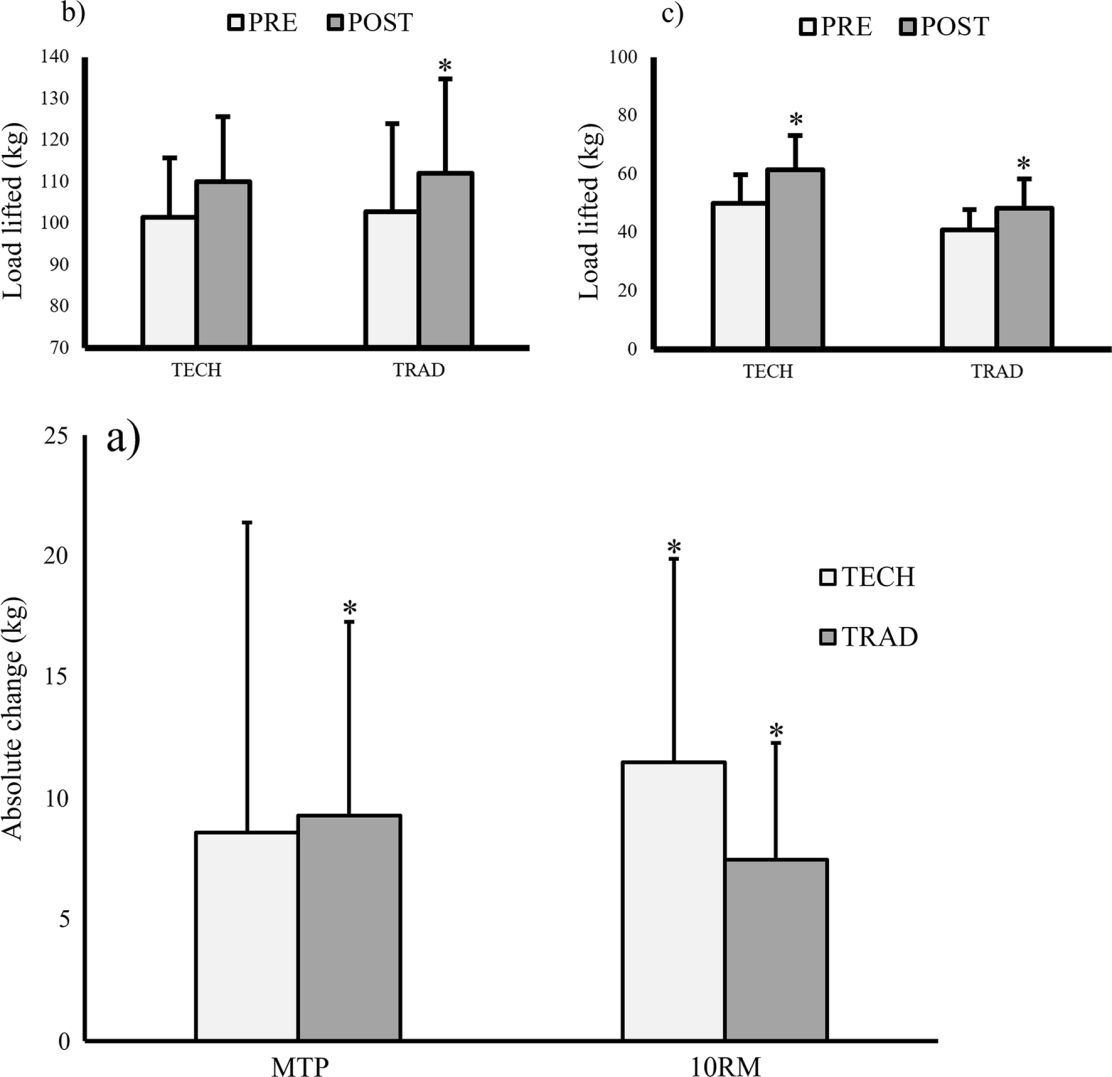
**a** Absolute change (kg) in the mid-thigh pull (MTP) and back squat 2_RIR_ with insets of the pre- and post-values for **b** the MTP and **c** the back squat 2_RIR_. * = Significant change from pre- to post-test (*p* < 0.01)

The 3_RIR_ load at pre-test was higher (ES = 1.11, *p* = 0.032) in the TECH group (50.0 ± 9.72 kg) compared to the TRAD group (40.83 ± 6.96 kg). Both the TECH (ES = 1.31, *p* = 0.002) and TRAD groups (ES = 1.48, p = 0.002; Fig. 4) improved their 3_RIR_ load, and when adjusting for the pre-test results, the 3_RIR_ load at post-test was not different between the groups (ES = 0.55, F = 1.083, *p* = 0.313).

No difference between the groups was found for isometric mid-thigh pull strength at pre-test (ES = 0.10, *p* = 0.872). The TRAD group (ES = 1.11, *p* = 0.008), but not the TECH group (ES = 0.64, *p* = 0.063) increased their isometric mid-thigh pull strength from pre- to post-test, and the post-test results were not different after adjusting for the pre-test results (F = 0.031, *p* = 0.863).

### Force distribution

The force distribution between the legs at pre-test was not different between the groups at most measuring points (*p* = 0.058–0.258), but the TRAD group displayed a larger difference in repetitions 9 + 10 in the 20 kg condition (ES = 0.92, *p* = 0.022), and in repetitions 1 + 2 in the RPE ≥ 7 condition (ES = 1.27, *p* = 0.016; Table 3). No change occurred from pre- to post-test in either group (*p* = 0.062–0.993), and the force distribution was similar between groups at all measuring points (F = 0.089–4.136, *p* = 0.098–0.488) except repetitions 9 + 10 in the RPE ≥ 7 condition (ES = 1.63, F = 8.506, *p* = 0.012).

**Table 3:** Force distribution difference (% difference between legs) during the first two (1 + 2) and last two repetitions (9 + 10) at the different loads

	Technological feedback group				Traditional feedback group			
	Pre-test	Post-test	Change	*p*	Pre-test	Post-test	Change	*p*
20 kg								
1 + 2	3.33 ± 2.53	2.33 ± 1.53	− 0.99 ± 2.23	0.190	4.87 ± 2.79	4.14 ± 5.18	− 0.73 ± 3.70	0.572
9 + 10	1.72 ± 1.41	2.14 ± 2.28	0.41 ± 1.89	0.506	4.76 ± 3.51*	4.15 ± 4.42	− 0.60 ± 2.91	0.552
50 kg								
1 + 2	2.36 ± 2.38	3.58 ± 2.41	1.21 ± 2.18	0.112	3.82 ± 3.09	4.71 ± 4.39	0.89 ± 3.49	0.467
9 + 10	2.08 ± 2.72	1.96 ± 1.46	− 0.12 ± 2.11	0.867	5.22 ± 3.96	4.30 ± 3.94	− 0.92 ± 3.19	0.413
RPE ≥ 7								
1 + 2	1.59 ± 1.26	2.01 ± 1.11	0.42 ± 1.34	0.351	3.90 ± 2.39*	3.88 ± 4.53	− 0.01 ± 2.54	0.987
9 + 10	2.13 ± 1.99	1.46 ± 0.87	− 0.67 ± 2.08	0.335	3.34 ± 2.47	4.84 ± 4.09*	1.50 ± 4.12	0.308
RPE_post_								
1 + 2	•	1.55 ± 1.33	− 0.01 ± 1.85	0.818	•	4.24 ± 3.51	2.91 ± 3.37	0.689
9 + 10	•	1.62 ± 0.98	0.15 ± 1.94	0.993	•	6.32 ± 3.76	0.27 ± 1.73	0.062

The *p*-values indicate significant pre-to-post changes

Changes for RPE_post_ are calculated relative to on the RPE ≥ 7 condition at pre-test

* = significantly different from the other group (*p* < 0.05)

### Center of gravity

The CV for the center of gravity was not different between the groups at pre-test at any measuring points (*p* = 0.057–0.797) except in the X-axis in the 20 kg condition (*p* = 0.004; Table 4). No changes occurred from pre- to post-test using the absolute loads (20 kg, 50% of BM, and RPE ≥ 7) for any of the groups (*p* = 0.160–0.969). When comparing the RPE_post_ to the pre-test RPE ≥ 7 condition, however, both groups increased the CV for the center of gravity in the X-axis (*p* < 0.001 and *p* = 0.006 for the TECH and TRAD groups, respectively). When adjusting for the pre-test results, no differences between the groups were found at post-test (F = 0.204—0.630, *p* = 0.441–0.657).

**Table 4:** Movement variation in the X- and Y-axes presented as coefficient of variation ((SD/mean) * 100)

	Technological feedback group				Traditional feedback group			
	Pre-test	Post-test	Change	*p*	Pre-test	Post-test	Change	*p*
20 kg								
X-axis	4.13 ± 0.97	3.87 ± 1.11	− 0.26 ± 0.69	0.260	3.00 ± 0.24*	3.25 ± 0.73	0.25 ± 0.65	0.286
Y-axis	9.99 ± 1.64	9.49 ± 1.97	− 0.49 ± 1.69	0.379	8.52 ± 1.47	9.01 ± 2.44	0.49 ± 2.68	0.598
50%								
X-axis	3.99 ± 1.07	4.36 ± 1.57	0.36 ± 0.88	0.229	3.51 ± 0.52	3.50 ± 1.05	− 0.15 ± 1.11	0.969
Y-axis	9.66 ± 0.90	9.55 ± 1.97	− 0.11 ± 1.74	0.846	9.19 ± 1.61	8.77 ± 2.08	− 0.42 ± 1.61	0.457
RPE ≥ 7								
X-axis	4.22 ± 1.92	3.85 ± 0.85	− 0.36 ± 0.94	0.252	3.54 ± 0.42	3.15 ± 0.73	− 0.39 ± 0.76	0.160
Y-axis	9.79 ± 1.60	9.39 ± 1.96	− 0.40 ± 1.99	0.542	9.58 ± 1.82	9.13 ± 1.97	− 0.45 ± 2.67	0.626
RPE_post_								
X-axis	•	5.71 ± 0.81	1.64 ± 0.84	< 0.001	•	5.11 ± 1.15	1.52 ± 0.97	0.006
Y-axis	•	8.98 ± 1.55	− 0.87 ± 1.79	0.182	•	8.70 ± 1.51	− 0.79 ± 1.79	0.288

The *p*-values indicate significant pre-to-post changes

Changes for RPE_post_ are calculated relative to on the RPE ≥ 7 condition at pre-test

* = significantly different from the other group (*p* < 0.01)

## Discussion

This study aimed to compare the changes in strength and movement quality in the back squat following a five-week RT program with either technological, open-ended augmented feedback or traditional, verbal feedback. No between-groups differences were detected for any of the variables, and no changes from pre- to post-test were found for the objective movement quality measurements (center of gravity and force distribution between the legs) when lifting the same loads. However, the traditional feedback group improved in the subjectively rated movement quality measurement as assessed by the RT-experts, whereas the technological feedback group did not.

Due to the increase in strength, one might have expected that lifting the same absolute loads at post-test (i.e., lower percentage of maximal strength) would have resulted in objectively better movement quality. Still, the force distribution between legs and the variation in the center of gravity did not change after the training intervention for either group. However, when comparing the same RPE (i.e., the RPE ≥ 7 at pre-test and the RPE_post_ condition), both groups displayed higher variation in the center of gravity at post-test. This may reflect an increased confidence among the participants regarding their strength following the five weeks of familiarization with the back squat exercise, resulting in them underestimating the RPE and overestimating the RIR. As such, the actual load relative to maximal strength could have been at a higher percentage at post-test compared to pre-test, thereby providing a higher difficulty in maintaining a constant center of gravity. Conversely, as the expert ratings did not identify a reduced technique, the increased variation in the center of gravity could potentially reflect an improved movement degeneracy, which is the ability to perform a movement in varying ways without compromising function [25]. Importantly, the low intra-rater reliability should be considered when interpreting the results. However, the three experts displayed substantial agreement, indicating that the provided ratings were reliable.

The importance of evenly distributing weight between the legs has previously been highlighted [3] and the authors recommended that any excessive unevenness should be treated properly before engaging in higher intensity and volume RT. The force distribution in the current study was generally between one and four percent at pre-test, which is lower than the previously proposed 6% cut-off value between people with equal and unequal weight distributions [3]. Hence, the lack of changes from pre- to post-test for both groups could be a result of this parameter having little potential for improvement. Indeed, albeit inexperienced in heavy RT, many of the participants included in the present study already reported frequent participation in other activities and movements before and during the intervention.

This was, to the authors’ knowledge, the first study to examine the effects on bodily movement quality and strength using automated feedback in a period of RT. Previous cross-sectional studies have shown that automated feedback has been able to correct the execution of movements with varying levels of complexity [8–10]. Therefore, it may be surprising that the current study was unable to detect changes following five weeks of training. The lack of changes in movement quality in the experimental tests despite increased training resistance could indicate that the participants became dependent on the feedback to continuously correct the movement. In fact, several participants reported missing the feedback from the laser pointers when lifting without feedback. One may argue that the training without automated visual feedback (TRAD) was more specific to the testing procedures. The importance of specificity in development of back squat performance has been demonstrated previously [26]. It is possible that a brief period of familiarization to lifting without the automated feedback should have been provided to the TECH group before the post-test.

Interestingly, the findings contradict the hypothesis that the participants would be able to apply the interaction between the visual feedback and the feeling of a given movement to achieve improved movement quality when tested without the feedback [15]. It is possible that conducting the experimental tests with the automated feedback could have displayed improved objective and subjective measures of movement quality. However, this would have made comparisons between pre- and post-test problematic. Moreover, the aim of training with feedback for a period should be to prepare the trainee for training independently of the feedback after a period of learning the movement.

Importantly, no injuries occurred in this training intervention and both groups increased the training loads as well as strength measured in the 2_RIR_ back squat and the isometric mid-thigh pull. The technological feedback method likely requires less experience and knowledge from the trainer/instructor and can be limited to one-to-two weeks of introduction in how to use the laser pointers and the information they provide. Hence, it could be argued that the technological feedback method has the potential to be a useful and valuable tool in the first stages of RT for populations without a special need for supervision outside of a general introduction to the movement.

The reader should consider some potential limitations of this study when interpreting the findings. First, only young females without extensive RT experience were recruited in this study and the findings may not be generalizable to other populations. Further, the movement quality likely degrades with increasing relative resistance (% of 1RM). Hence, one should be cautious of transferring the current findings to other forms of RT or other complex exercises and movements. Moreover, the subjective measurements RIR and RPE measurements may have been inaccurate and influenced by the participants’ familiarization with the back squat training. It is important to note that RIR is not a direct measure of maximal strength or degree of fatigue. However, the sets were terminated at a high RPE, suggesting that they were performed to near failure. According to the findings of Zourdos and colleagues [16], the accuracy of the RIR method is highest close to failure (i.e., few RIR). Finally, the low sample size (n = 19) and low total training volume in this intervention (ten session over five weeks) must be acknowledged. Although this study provides novel insights into the potential effects of technological feedback, future research is needed to confirm these findings in larger populations and over longer time frames.

### Conclusion

In conclusion, five weeks of supervised RT with either traditional or technological feedback increased strength, but not objectively assessed indicators of movement quality in young females with little limited RT experience. The results suggest that beginner trainees can expect similar effects in the first five weeks of RT using either feedback method, and the choice of method should be based on the available expertise and equipment. This was the first study to examine the effects of training automated feedback using body-lights in the back squat and more research is needed to confirm the findings. Future studies should consider comparing the training effects with and without feedback during the experimental testing to address whether the different feedback methods influence the movement quality after familiarization with both the feedback and the movement.

## Data Availability

All relevant datasets are available upon reasonable request to the corresponding author.

## Abbreviations

ANCOVA: Analysis of covariance
BM: Body mass
ES: Effect size
RM: Repetitions maximum
RIR: Repetitions in reserve
RPE: Rate of perceived exertion
RT: Resistance-training
TECH: Technological feedback group
TRAD: Traditional feedback group
